# Elastic properties of Fe-bearing Akimotoite at mantle conditions: Implications for composition and temperature in lower mantle transition zone

**DOI:** 10.1016/j.fmre.2021.12.013

**Published:** 2022-01-21

**Authors:** Yajie Zhao, Zhongqing Wu, Shangqin Hao, Wenzhong Wang, Xin Deng, Jian Song

**Affiliations:** a Laboratory of Seismic and Physics of Earth's Interior, School of Earth and Space Sciences, University of Science and Technology of China, Hefei 230026, China; b National Geophysical Observatory at Mengcheng, University of Science and Technology of China, Hefei, China; c CAS Center for Excellence in Comparative Planetology, USTC, Hefei 230026, China; d Institute of Geophysics and Planetary Physics, Scripps Institution of Oceanography, University of California San Diego, La Jolla 92092, CA, USA; e Department of Earth Sciences, University College London, London WC1E 6BT, United Kingdom; f Earth and Planets Laboratory, Carnegie Institution for Science, Washington, DC 20015, USA

**Keywords:** Akimotoite, Pyrolite, Elastic moduli, Temperature heterogeneity, Gaussian distribution

## Abstract

The pyrolite model, which can reproduce the upper-mantle seismic velocity and density profiles, was suggested to have significantly lower velocities and density than seismic models in the lower mantle transition zone (MTZ). This argument has been taken as mineral-physics evidence for a compositionally distinct lower MTZ. However, previous studies only estimated the pyrolite velocities and density along a one-dimension (1D) geotherm and never considered the effect of lateral temperature heterogeneity. Because the majorite-perovskite-akimotoite triple point is close to the normal mantle geotherm in the lower MTZ, the lateral low-temperature anomaly can result in the presence of a significant fraction of akimotoite in pyrolitic lower MTZ. In this study, we reported the elastic properties of Fe-bearing akimotoite based on first-principles calculations. Combining with literature data, we found that the seismic velocities and density of the pyrolite model can match well those in the lower MTZ when the lateral temperature heterogeneity is modeled by a Gaussian distribution with a standard deviation of ∼100 K and an average temperature of dozens of K higher than the triple point of MgSiO_3_. We suggest that a harzburgite-rich lower MTZ is not required and the whole mantle convection is expected to be more favorable globally.

## Graphical abstract

After considering Gaussian temperature distribution, the velocities and density of the pyrolite model fit the seismic velocity profile well in the lower MTZ because of the presence of akimotoite. A lower MTZ with a composition significantly different from the pyrolite model is not requisite.

**Figure:**
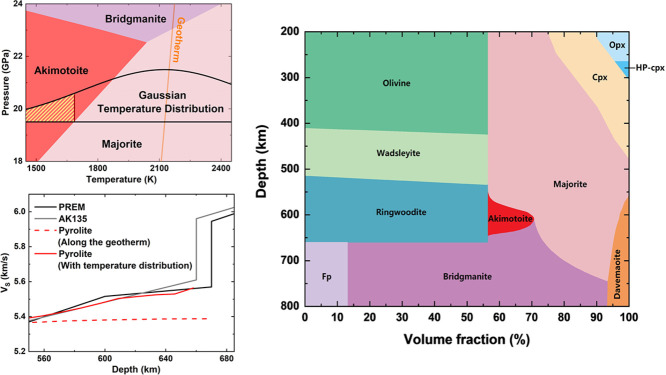

## 1 Introduction

The pyrolite model proposed by Ringwood [1] has been widely used as a reference for the upper-mantle composition. Previous studies suggested that the velocities and density of the pyrolite model can match the one-dimension (1D) seismological models such as PREM [2] and AK135 [3] for the upper mantle [4] and the lower mantle [5,6]. However, the shear wave velocity and density of the pyrolite model are lower than PREM or AK135 up to 4% and 2% in the lower mantle transition zone (MTZ) [7], [8], [9]. Based on the complicated slab dynamics in the lower MTZ, different hypotheses including the enrichment of harzburgite-rich materials were introduced to reconcile this discrepancy [8,9].

**Fig. 1:**
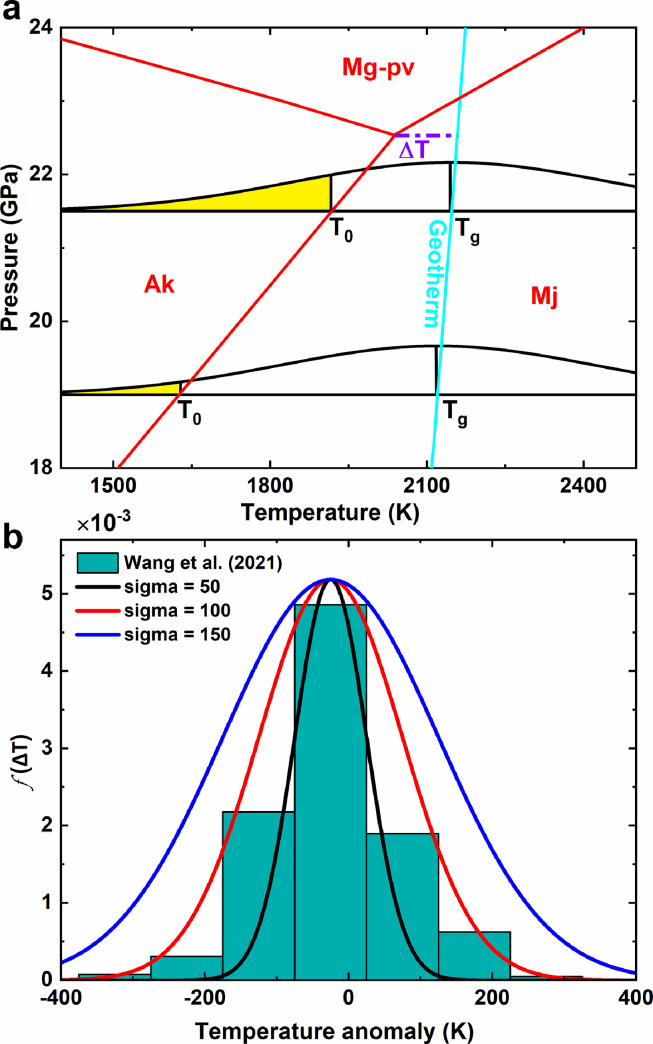
The lateral temperature distribution in the lower MTZ. (a) Schematic diagram of calculating volume proportion of akimotoite by MgSiO_3_ phase diagram with Gaussian distribution. Red lines are the phase boundary from Ishii et al. [17]. The cyan line represents the normal mantle geotherm. Black lines represent the Gaussian distribution of temperature at two depths. The yellow shaded parts represent the temperature where akimotoite exists. *ΔT* is the temperature difference between geotherm $T_{g}$ and triple point of the MgSiO_3_ phase transition. (b) Comparison of distributions of temperature anomaly from Wang et al. [11] and Gaussian distribution of different σ.

The velocities and density of the pyrolite model in previous studies were only estimated along the 1D geotherm. The temperature lateral heterogeneity clearly indicated by the seismic tomography model [10], [11], [12], [13] is ignored in the calculations. In general, this neglection will not affect the estimates of velocities and density because the velocities and density of minerals both depend near-linearly on the temperature. However, when the temperature heterogeneity can affect the mineral phases, the conventional method to calculate the velocities and density of the pyrolite model along the geotherm becomes inappropriate because this method did not include the effect of the phase transition on the velocities and density. The lower MTZ, where the calculated velocities and density of the pyrolite model using the conventional method fail to match the PREM and AK135 model, locates just the depth range where the temperature heterogeneity can significantly change the mineral phases in the pyrolite model (Fig. 1a). The majoritic garnet-akimotoite and akimotoite-bridgmanite phase transition occur at ∼21-23 GPa and ∼23-27 GPa [14], [15], [16] respectively, which correspond to the depths of the lower MTZ. The phase transition temperatures are close to the normal geotherm [17], [18], [19]. If low-temperature anomalies exist laterally, the pyrolite model will be expected to consist of a significant fraction of akimotoite. Can a pyrolite composition explain the velocities and density of the lower MTZ after including the mineral phase variations? Does mineral physics require a harzburgite-rich lower MTZ?

The answers to the questions, which have fundamental implications to the mantle convection, require the elastic properties of iron-bearing akimotoite at high pressures and temperatures. The equation of states and elastic properties of MgSiO_3_ akimotoite have been investigated by previous studies [20], [21], [22], [23], [24], [25], [26], [27], [28]. The sound velocities of the iron-bearing akimotoite (Mg_0.9_,Fe_0.1_)SiO_3_ have been measured by Siersch [9] up to 26 GPa and 1100 K using ultrasonic interferometry and synchrotron X-ray diffraction. However, the velocities and density of iron-bearing akimotoite at MTZ conditions remain unknown.

In this study, we investigated the elastic properties of $(Mg_{0.875},Fe_{0.125})\text{Si}O_{3}$ akimotoite at mantle conditions using first-principles calculations. The calculated results are consistent with the available experimental data. The temperature heterogeneity in the lower MTZ will affect the volume proportion of akimotoite and hence the velocities and density of the pyrolite model. Combining our results with the elastic properties of other minerals, we calculated the velocities and density of the pyrolite model in the lower MTZ with the effect of temperature heterogeneity and compared mineral-physics results with seismic reference models.

## 2 Material and methods

### 2.1 First-principles calculations

The calculations were performed using the Quantum Espresso package [29] based on the density functional theory (DFT) [30]. The local density approximation (LDA) was adopted for exchange-correlation potential [31,32]. The pseudopotential for magnesium was generated by von Barth and Car [33], and the iron pseudopotential was generated using the method of Vanderbilt [34]. The pseudopotentials for oxygen and silicon were generated by Troullier and Martins [35]. The plane wave kinetic energy and charge density cutoff were 70 Ry and 700 Ry, respectively. The initial structure for $(Mg_{1-x},Fe_{x})\text{Si}O_{3}$ akimotoite with x=0.125 (akimotoite refers to the composition of $(Mg_{0.875},Fe_{0.125})\text{Si}O_{3}$ hereafter unless otherwise specified) is generated by replacing a Mg atom with iron in a 40-atoms unit cell. The akimotoite structures at different pressures were optimized with a 2×2×2 k-point mesh by using damped variable cell shape molecular dynamics [36]. The dynamical matrices were calculated using the density functional perturbation theory (DFPT) [37] to obtain vibrational frequencies at a 2×2×2 q-point mesh for each optimized structure.

### 2.2 Elasticity and anisotropy calculations

Isothermal elastic tensors are determined by second derivative of free energy with strain from Eq. 1 [38]:

(1) $$C_{ijkl}^{T}=\frac{1}{V}\left(\frac{\partial^{2}F}{\partial e_{ij}\partial e_{kl}}\right)+\frac{1}{2}P\left(2\delta_{ij}\delta_{kl}-\delta_{il}\delta_{kj}-\delta_{ik}\delta_{jl}\right)$$

Helmholtz free energy at volume *V* with infinitesimal strains *e_ij_* (i,j=1,2,3) can be obtained from the quasi-harmonic approximation:

(2) $$F\left(V,T,e_{ij}\right)=U_{0}\left(V,e_{ij}\right)+\sum_{q,m}\frac{\hbar \omega_{qm}\left(V,e_{ij}\right)}{2}+K_{B}T\sum_{q,m}ln\left\{1-exp\left[-\frac{\hbar \omega_{qm}\left(V,e_{ij}\right)}{k_{B}T}\right]\right\}$$

The $\omega_{q,m}$ represents vibrational frequency of the normal mode *m* at phonon wave vector *q*. ℏ and $K_{B}$ are the Planck and Boltzmann constants, respectively. Thus, the conventional method for elastic properties calculations requires vibrational frequencies at numerous strained configurations, which need a large amount of computation. In this study, we adopted the method proposed by Wu and Wentzcovitch [39], which only requires vibrational density of states of unstrained configurations and elastic tensors at static conditions. This method reduces the computation to one tenth of the conventional method and maintains accuracy. The method has been successfully applied to many minerals [4,26,[40], [41], [42], [43], [44], [45]].

Based on Kelvin-Christoffel equation, we can obtain the wave velocities propagating in different directions:

(3) $$\left|C_{\text{ijkl}}n_{j}n_{l}-\rho V^{2}\delta_{ik}\right|=0$$

where n=(n_1_, n_2_, n_3_) is the vector of propagation direction, $C_{ijkl}$ is the four-order elastic tensor, ρ presents the density, and V is the wave velocity. The P wave anisotropy $\left(A_{P}\right)$, S wave anisotropy $\left(A_{S}\right)$, and maximum polarization anisotropy ($A_{S}^{po}$) are defined as:

(4a) $$A_{P}=2\times \frac{\left(V_{P}^{max}-V_{P}^{min}\right)}{\left(V_{P}^{max}+V_{P}^{min}\right)}$$

(4b) $$A_{S}=2\times \frac{\left(V_{S}^{max}-V_{S}^{min}\right)}{\left(V_{S}^{max}+V_{S}^{min}\right)}$$

(4c) $$A_{S}^{po}=2\times \frac{{\left(V_{S1}-V_{S2}\right)}_{max}}{\left(V_{S1}+V_{S2}\right)}$$

### 2.3 Geophysical modeling

The pyrolite model along the normal geotherm contains ∼56 vol.% ringwoodite ((Mg,Fe)_2_SiO_4_), ∼39 vol.% majoritic garnet ((Mg,Fe,Ca)_3_(Mg,Si,Al)_2_Si_3_O_12_), and ∼5 vol.% Davemaoite (CaSiO_3_) in the lower MTZ [46], [47], [48], [49], [50]. The Fe/(Fe+Mg) of the pyrolite model is ∼11 mol.% [51]. The iron partition coefficients $K_{D}$ of garnet-ringwoodite, akimotoite-ringwoodite, ringwoodite-ferropericlase, and bridgmanite-ferropericlase are around 0.8, 0.4, 0.65, and 0.7, respectively [14,17,[52], [53], [54]]. Based on the total iron content in the pyrolite model and partition coefficients between different minerals, the Fe/(Fe+Mg) in ringwoodite, garnet, akimotoite, and bridgmanite is ∼10 mol.%, ∼8 mol.%, ∼4 mol.%, and ∼10 mol.% respectively. The iron content of the majoritic garnet estimated in this study is closed to those from Irifune et al. [7] (∼7 mol.%). Based on the chemical composition of garnet (pyrolite minus olivine) from Irifune et al. [7], the majoritic garnet is Py_15_Gr_26_Alm_7_Mj_52_ with ∼15 vol.% pyrope [40], ∼26 vol.% grossular [4], ∼7 vol.% almandine [55], and ∼52 vol.% majorite at 20 GPa, which is similar to Arimoto et al. [55] and Pamato et al. [8]. The chemical composition of majoritic garnet varies with depth mainly because of the exsolution of Davemaoite. In the lower MTZ, the majoritic garnet is Py_31_Gr_10_Alm_8_Mj_51_ since there is ∼5 vol.% Davemaoite transformed from majoritic garnet. The elastic modulus and density of the pyrolite model are calculated using Eqs. 5, 6 [56]:

(5) $$M=\left[\sum_{i}f_{i}M_{i}+{\left(\sum_{i}f_{i}M_{i}^{-1}\right)}^{-1}\right]/2$$

(6) $$\rho =\sum_{i}f_{i}\rho_{i}$$

where $f_{i}$, $\rho_{i}$, and $M_{i}$ are the volume fraction, density, and moduli of the ith mineral, respectively.

The temperature lateral heterogeneity will change the mineral phase in the lower MTZ. To investigate this effect on the velocities and density of the pyrolite model, we assume a Gaussian temperature distribution for the temperature heterogeneity of an entire spherical layer at a certain depth:

(7) $$f\left(T;T_{g},\sigma \right)=\frac{1}{\sigma \sqrt{2\pi }}\text{exp}\left(-\frac{{\left(T-T_{g}\right)}^{2}}{2\sigma^{2}}\right)$$

Here, *T_g_* is the normal geothermal temperature and σ is the standard deviation of mantle temperature. The ratio of akimotoite is determined by

(8) $$y\left(T_{0};T_{g},\sigma \right)=\frac{1}{\sigma \sqrt{2\pi }}\int_{-\infty }^{T_{0}}\text{exp}\left(-\frac{{\left(T-T_{g}\right)}^{2}}{2\sigma^{2}}\right)dT$$

Here, *T_0_* is temperature at which garnet transforms to akimotoite or akimotoite transforms to bridgmanite (Fig. 1a). The degree to which the pyrolite model matches the $V_{P}$, $V_{S}$ and density is evaluated using the misfit function $D_{all}$:

(9) $$D_{all}=\sqrt{\frac{\sum_{i=1}^{n}\left({\left(\frac{V_{Pi}^{py}}{V_{Pi}^{model}}-1\right)}^{2}+{\left(\frac{V_{Si}^{py}}{V_{Si}^{model}}-1\right)}^{2}+{\left(\frac{\rho_{i}^{py}}{\rho_{i}^{model}}-1\right)}^{2}\right)}{3n}}$$

where $V_{Pi}^{py}$, $V_{si}^{py}$, $\rho_{i}^{py}$, $V_{Pi}^{model}$, $V_{si}^{model}$ and $\rho_{i}^{model}$ represent the P- and S-wave velocities and density of the pyrolite model and seismic models, respectively, and *n* is the number of data points.

## 3 Results and discussion

### 3.1 Elastic properties of akimotoite at high pressures and temperatures

**Fig. 2:**
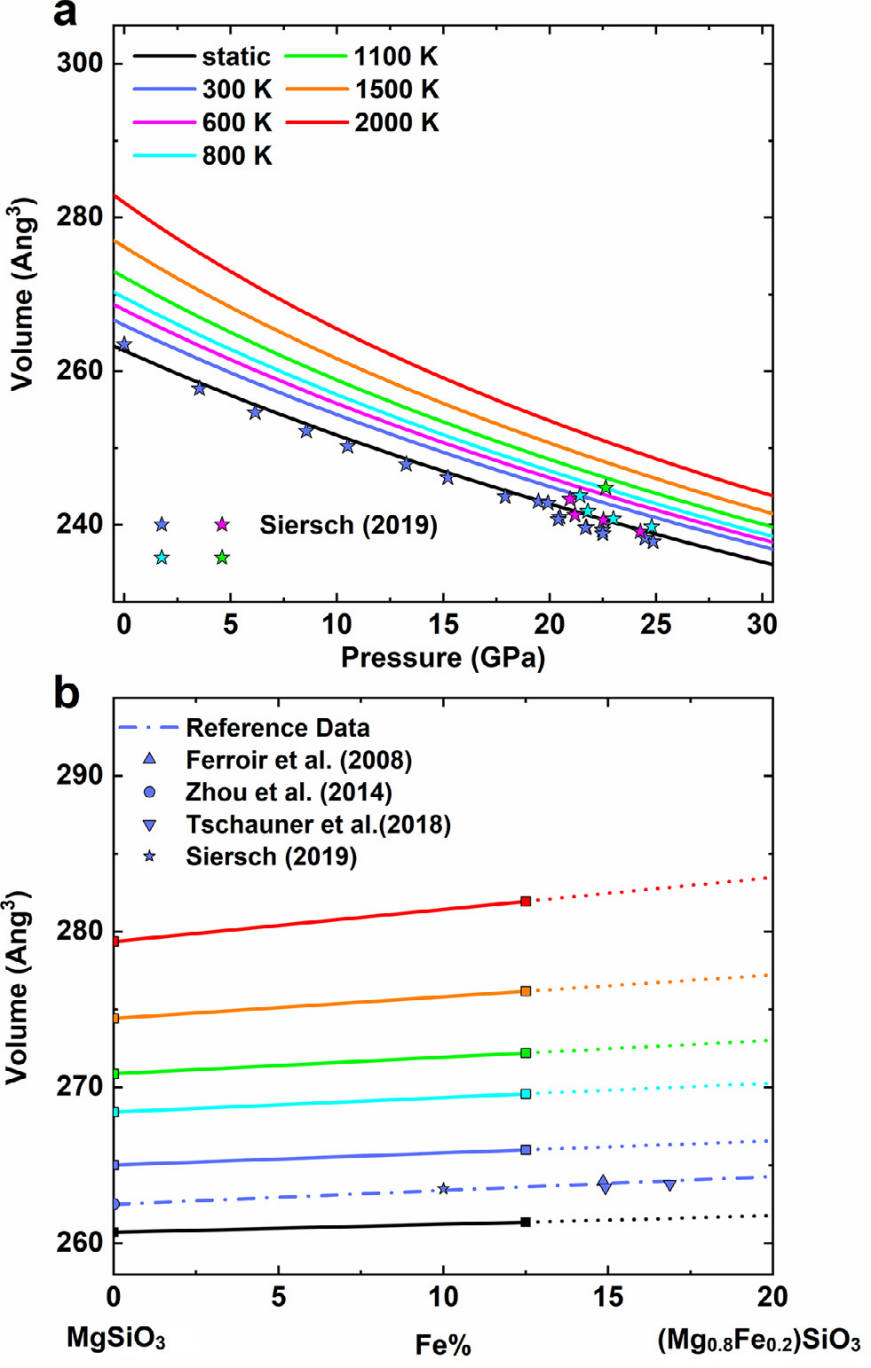
**The volumes of akimotoite at various pressures, temperatures, and Fe content.** (a) Equation of states of akimotoite. The solid lines represent our calculation results for Mg_0.875_Fe_0.125_SiO_3_, and the solid scatters are for Mg_0.9_Fe_0.1_SiO_3_ from Siersch [9]. (b) Effect of Fe content on volume of akimotoite in different temperatures at 0 GPa. Solid lines are fitted by volume of MgSiO_3_ akimotoite [26] and Mg_0.875_Fe_0.125_SiO_3_ (this study), and the dotted lines represent the extrapolation. Dot-dashed line is fitted by experiment data [9,20,57,58].

The equation of states of akimotoite up to 30 GPa and 2000 K are shown in Fig. 2a and listed in Table 1. The calculated volume at ambient temperature is ∼1.5% larger than the previous experimental measurements [9]. It was observed that such discrepancies between calculations and experiments dwindled away while the pressure and the temperature increased. At MTZ's pressure, the relative differences of volume are less than 0.5% at 1100 K. The volume of akimotoite increases linearly with iron concentration [9,20,57,58]. The effect of iron content on volume (Fig. 2b), which is estimated based on MgSiO_3_ akimotoite from Hao et al. [26] and (Mg_0.875_,Fe_0.125_)SiO_3_ akimotoite in this study, agrees well with the experimental data at ambient conditions.

**Table 1:** Equation of state parameters of akimotoite at 0 GPa.

Reference	Composition	T (K)	V_0_ (Å^3^)	K_T_ (GPa)	${\left(\partial K_{T}/\partial P\right)}_{T}$
This study	Mg_0.875_Fe_0.125_SiO_3_	Static	262.7	213.2	4.35
This study	Mg_0.875_Fe_0.125_SiO_3_	300	266.0	202.4	4.49
This study	Mg_0.875_Fe_0.125_SiO_3_	1000	271.3	180.1	4.77
This study	Mg_0.875_Fe_0.125_SiO_3_	2000	282.0	141.3	5.40
Seirsch (2019)	Mg_0.9_Fe_0.1_SiO_3_	300	263.5	197	5.3
Hao et al. (2019)	MgSiO_3_	300	265.2	202	4.40
Seirsch et al. (2021)	MgSiO_3_	300	262.43	205	4.9

K_T_: the isothermal bulk modulus at 0 GPa.

**Fig. 3:**
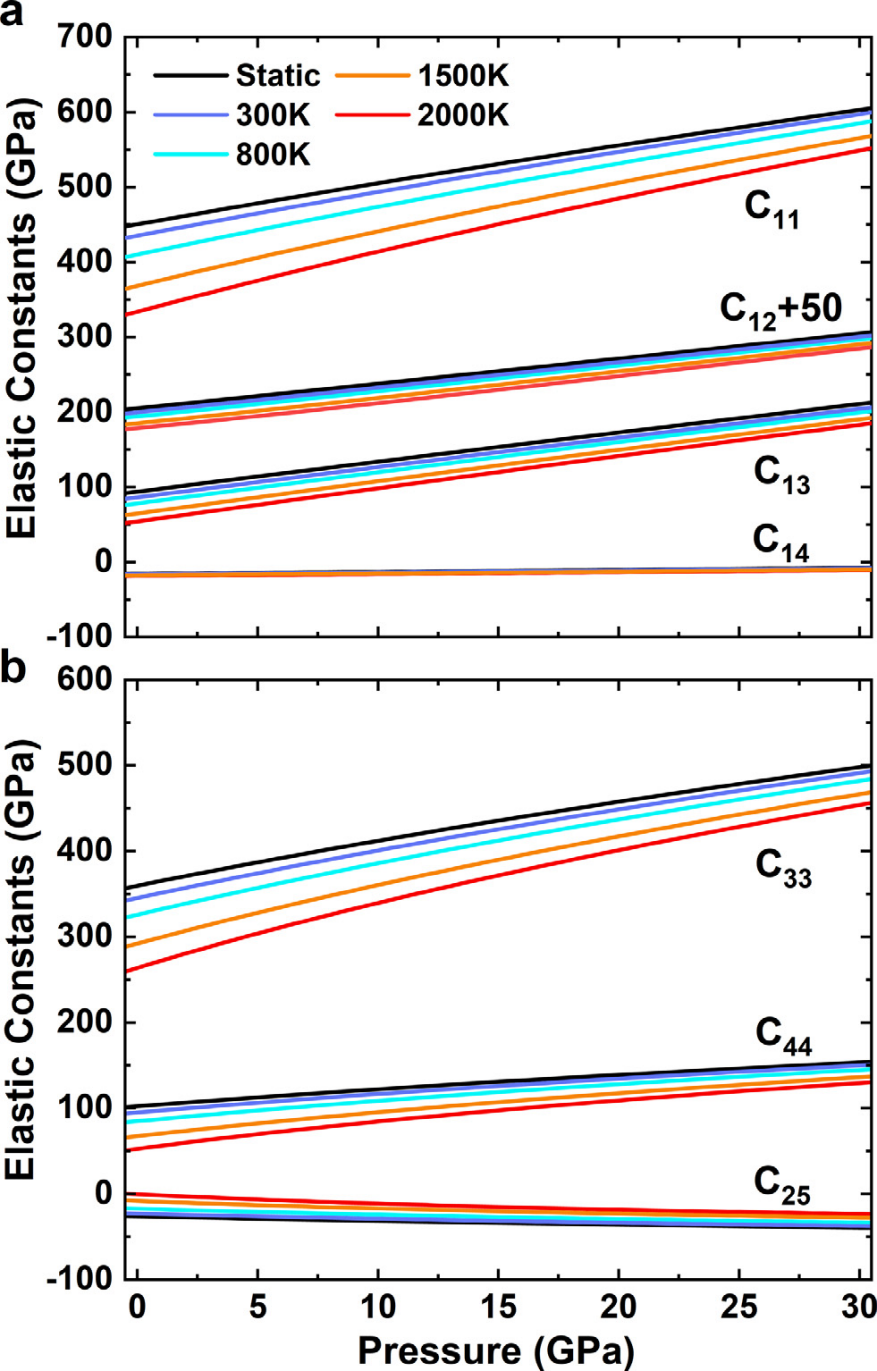
Pressure dependences of elastic constants of akimotoite at various temperatures.

**Fig. 4:**
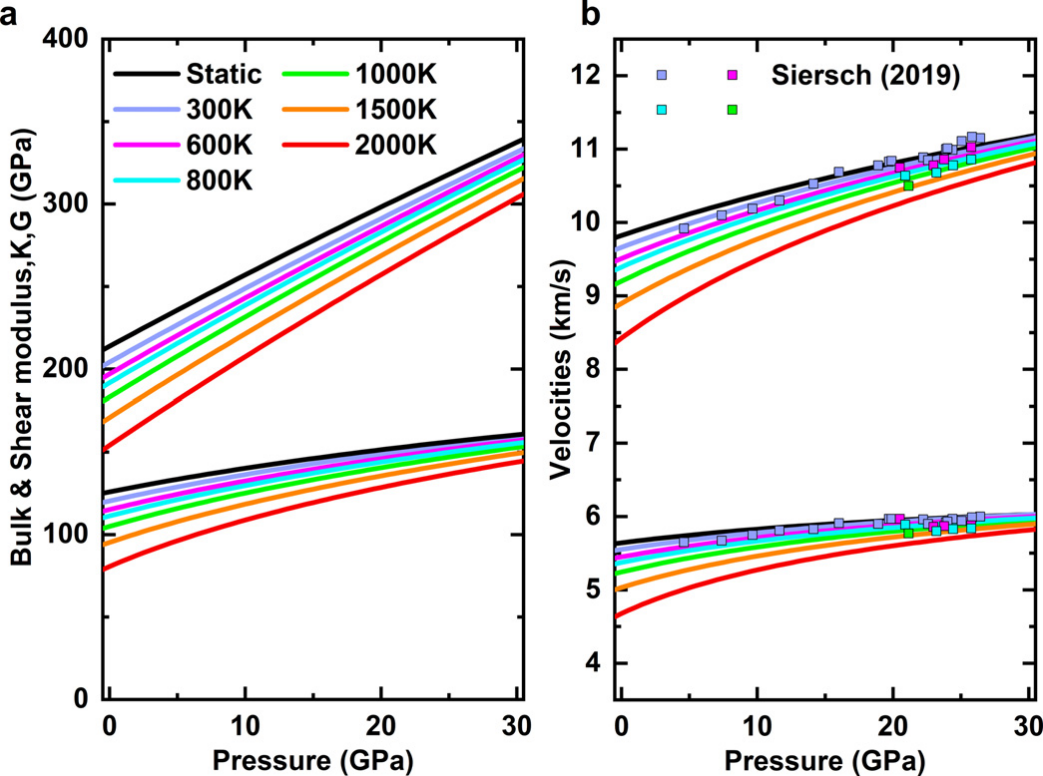
Pressure dependences of (a) bulk and shear modules and (b) velocities at various temperatures. Experimental data source: squares, Siersch [9].

Akimotoite has a trigonal structure, and its elastic tensor can be determined by seven independent parameters C_11_, C_12_, C_13_, C_14_, C_25_, C_33_, and C_44_ (Fig. 3, Table S1 and S2). The adiabatic bulk moduli K_S_ and shear moduli G are calculated based on the Voigt-Reuss-Hill average [56] (Fig. 4a). The derivatives of elastic modulus with respect to *PT* are listed in Tables S1 and S2. The compression wave velocity $V_{P}$ and shear wave velocity $V_{S}$ are given by $V_{P}=\sqrt{\frac{K_{S}+\frac{4}{3}G}{\rho }}$ and $V_{S}=\sqrt{\frac{G}{\rho }}$, and their derivatives with respect to *PT* are shown in Fig. 4b and Table S3. The calculated wave velocities agree with those measured by Siersch [9] within ∼1%.

The pressure dependences of anisotropies at various temperatures are shown in Fig. S1. The anisotropy of MgSiO_3_ akimotoite is larger than other major minerals in the lower MTZ [26,27]. Our calculation shows that iron further increases the anisotropies of akimotoite. At 20 GPa and 1500 K, $A_{P}$, $A_{S}$, and $A_{S}^{PO}$ of iron-bearing akimotoite are 2.2%, 6.4%, and 6.1% larger than MgSiO_3_ akimotoite respectively. The differences of $A_{P}$, $A_{S}$, and $A_{S}^{PO}$ between iron-bearing and MgSiO_3_ akimotoite increase to 2.4%, 7.7%, and 7.4% respectively at 24 GPa and 2000 K.

### 3.2 The pyrolite model can account for the velocities and density of the lower MTZ

**Fig. 5:**
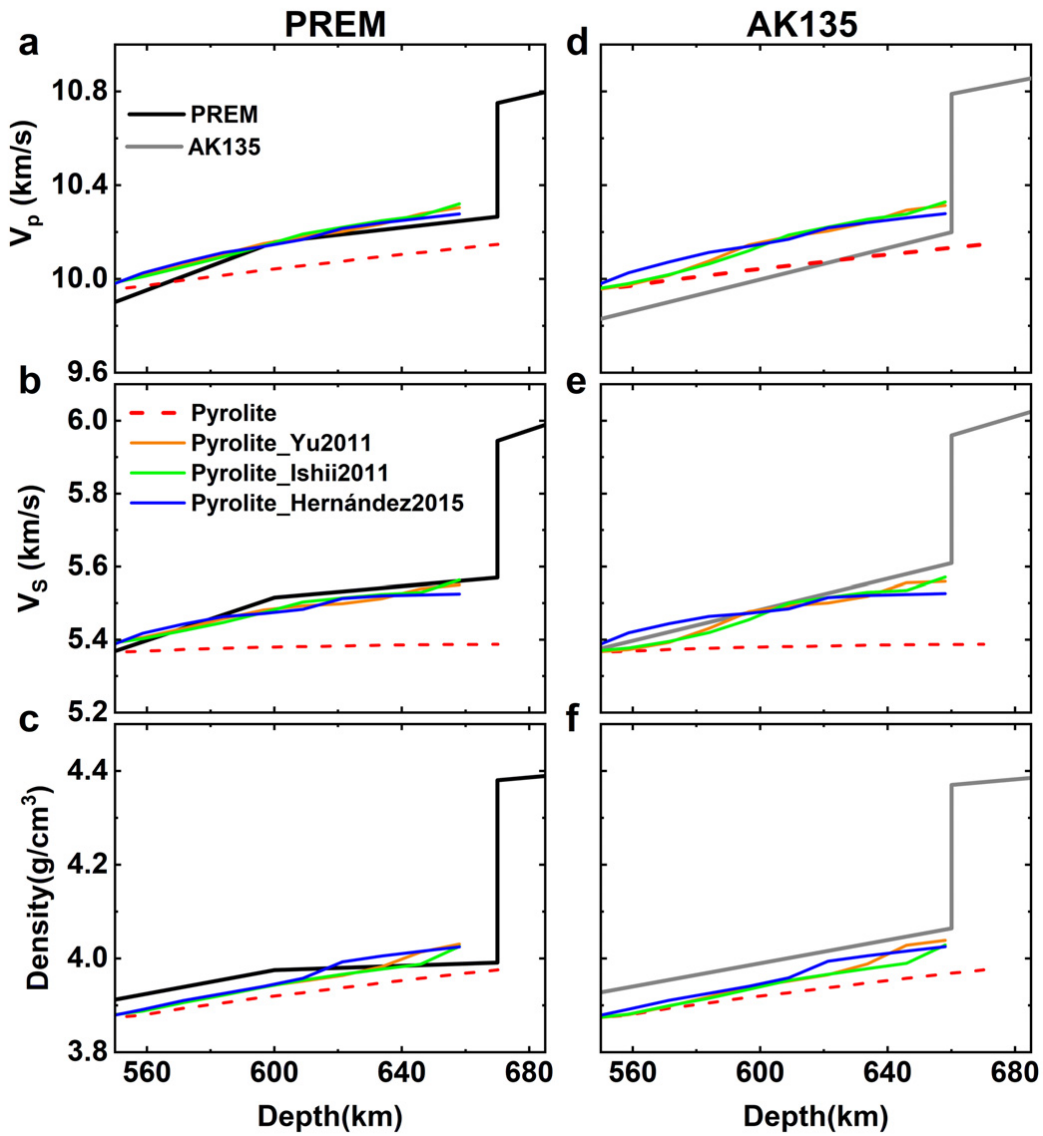
(a and d) Compressional wave velocities *V_P_*, (b and e) shear wave velocities V_S_, and (c and f) densities of pyrolite model and seismic models. The black and grey lines represent PREM [2] and AK135 [3] model respectively. The red dashed lines represent pyrolite along the normal geotherm. The orange, green and blue lines are the results of pyrolite with temperature heterogeneity corresponding to the smallest misfit functions $D_{all}$ based on the phase boundary of Yu et al. [19], Ishii et al. [17], and Hernández et al. [18] respectively. (a-c) are the fitting results based on PREM and (d-f) are the fitting results based on AK135.

Combining the elasticities of akimotoite and other minerals in the lower MTZ (summarized in Table 2), we calculated the velocities and density of the pyrolite model along the normal geotherm. As shown in Fig. 5, the S-wave velocity of the pyrolite model in the lower MTZ is lower than PREM or AK135 up to 0.2 km/s, similar to previous studies [7,8]. The stagnant slabs in the lower MTZ may contain non-negligible harzburgite. However, the velocities of harzburgite with an initial composition of 81.5 vol.% wadsleyite and 18.5 vol.% majoritic garnet are still lower than the PREM or AK135 ∼0.1 km/s unless the temperature in the lower MTZ is 200 K lower than the adiabat with a potential temperature of 1673 K [8]. The stable field of akimotoite is close to the mantle geotherm. The harzburgite mineral model can contain ∼15 vol.% akimotoite at the temperature around 1673-1873 K [59,60]. Siersch [9] found that the velocities of harzburgite can match PREM and AK135 after including the contribution of the akimotoite phase. Their results suggested a pure harzburgite and a reference temperature below 1873 K for the lower MTZ. Although the presence of a harzburgite-rich layer at local regions is supported by the stagnant slabs in the lower MTZ, the mechanism for a global harzburgite layer in the lower MTZ remains unclear.

**Table 2:** Bulk (Ks) and Shear (G) Moduli of minerals at base of the transition zone employed in the calculation of density and seismic velocities.

**Composition**	***K_S_ (GPa)***	$\partial K_{S}/\partial P$	$\partial K_{S}/\partial T$*(MPa/K)*	$\partial^{2}K_{S}/\partial P^{2}$$\left(\times 10^{-3}\mathrm{GP}a^{-1}\right)$	$\partial^{2}K_{S}/\partial P\partial T$$\left(\times 10^{-4}K^{-1}\right)$	$\partial^{2}K_{S}/\partial T^{2}$$\left(\times 10^{-6}\mathrm{GPa}/K^{2}\right)$	Ref
**Ringwoodite**							
Mg_2_SiO_4_	184.9	4.23	-15.05	-8.25	0.54	-0.70	Cal.1
(Mg_0.875_Fe_0.125_)_2_SiO_4_	186.9	4.79	-20.99	-21.61	3.43	-1.94	Cal.1
**Garnet**							
Mg_3_Al_2_Si_3_O_12_	176.3	3.79	-5.59	1.36	-8.88	4.85	Cal.2
Ca_3_Al_2_Si_3_O_12_	170.0	4.22	-8.01	-4.96	-1.70	0.48	Cal.3
Fe_3_Al_2_Si_3_O_12_	172.6	4.43	-18.7				Exp.1
Mg_4_Si_4_O_12_	159.7	4.64	-14.70	-13.73	0.47	-0.27	
**Akimotoite**							
MgSiO_3_	204.9	4.45	-17.72	-11.39	1.49	-1.18	Cal.4
Mg_0.875_Fe_0.125_SiO_3_	205.1	4.62	-24.07	-13.71	4.35	-2.86	This study
**Ca-perovskite**							
CaSiO_3_	248	4.2	-36				Exp.2
**Bridgmanite**							
MgSiO_3_	247.5	4.00	-19.38	-4.96	1.03	-1.42	Cal.5
Mg_0.875_Fe_0.125_SiO_3_	249.4	4.04	-19.56	-5.07	0.96	-1.37	Cal.5

	***G (GPa)***	∂G/∂P	∂G/∂T***(MPa/K)***	$\partial^{2}G/\partial P^{2}$$\left(\times 10^{-3}\mathrm{GP}a^{-1}\right)$	$\partial^{2}G/\partial P\partial T$$\left(\times 10^{-4}K^{-1}\right)$	$\partial^{2}G/\partial T^{2}$$\left(\times 10^{-6}\mathrm{GPa}/K^{2}\right)$	

**Ringwoodite**							
Mg_2_SiO_4_	120.9	1.30	-10.96	-16.73	1.72	-0.39	Cal.1
(Mg_0.875_Fe_0.125_)_2_SiO_4_	115.2	1.35	-13.06	-20.71	3.06	-1.02	Cal.1
**Garnet**							
Mg_3_Al_2_Si_3_O_12_	93.95	1.35	-6.48	-13.54	-3.76	2.76	Cal.2
Ca_3_Al_2_Si_3_O_12_	106.3	1.29	-6.03	-14.7	0.07	0.38	Cal.3
Fe_3_Al_2_Si_3_O_12_	94.2	1.06	-12.6				Exp.1
Mg_4_Si_4_O_12_	83.53	1.28	-8.77	-14.19	0.97	-0.01	
**Akimotoite**							
MgSiO_3_	128.1	1.79	-13.77	-17.13	2.26	-0.81	Cal.4
Mg_0.875_Fe_0.125_SiO_3_	120.9	1.85	-17.58	-20.99	4.58	-2.28	This study
**Ca-perovskite**							
CaSiO_3_	126	1.6	-15				Exp.2
**Bridgmanite**							
MgSiO_3_	167.6	1.79	-19.8	-7.82	1.81	-1.21	Cal.5
Mg_0.875_Fe_0.125_SiO_3_	164.6	1.79	-19.74	-8.34	1.85	-1.20	Cal.5

Cal.1 Núñez Valdez et al. [63], Cal. 2 Hu et al. [38], Cal. 3 Duan et al. [4], Cal. 4 Hao et al. [26], Cal.5 Shukla et al. [64], Exp. 1 Arimoto et al. [53], Exp. 2 Greaux et al. [65] . The data from calculations are fitted for the pressure and temperature range of 0-30 GPa and 270-2000 K respectively based on the equation: $M=M_{0}+\frac{\partial M}{\partial P}\times P+\frac{\partial M}{\partial T}\times \left(T-270\right)+\frac{\partial^{2}M}{\partial P^{2}}\times P^{2}+\frac{\partial^{2}M}{\partial P\partial T}\times P\times \left(T-270\right)+\frac{\partial^{2}M}{\partial T^{2}}\times {\left(T-270\right)}^{2}$. M represents the elastic moduli. P is pressure in GPa, and T is temperature in Kelvin.

The above-mentioned velocities and density of pyrolite and harzburgite along geotherm were calculated based on the average temperature and the mineral phases at the average temperature. In general, ignoring the secular temperature distribution in Earth's interior does not affect the 1D velocity and density profiles since they depend near-linearly on temperature. However, in the lower MTZ where the stable field of akimotoite is close to the mantle geotherm, akimotoite can appear even geotherm is above the stable field of akimotoite and the akimotoite fraction is sensitive to the temperature distribution (Fig. 1a). Since akimotoite has much higher velocities than majoritic garnet especially $V_{S}$ (Fig. S2) [26,27], the effect of akimotoite on the velocities and density of the pyrolite model cannot be ignored.

**Fig. 6:**
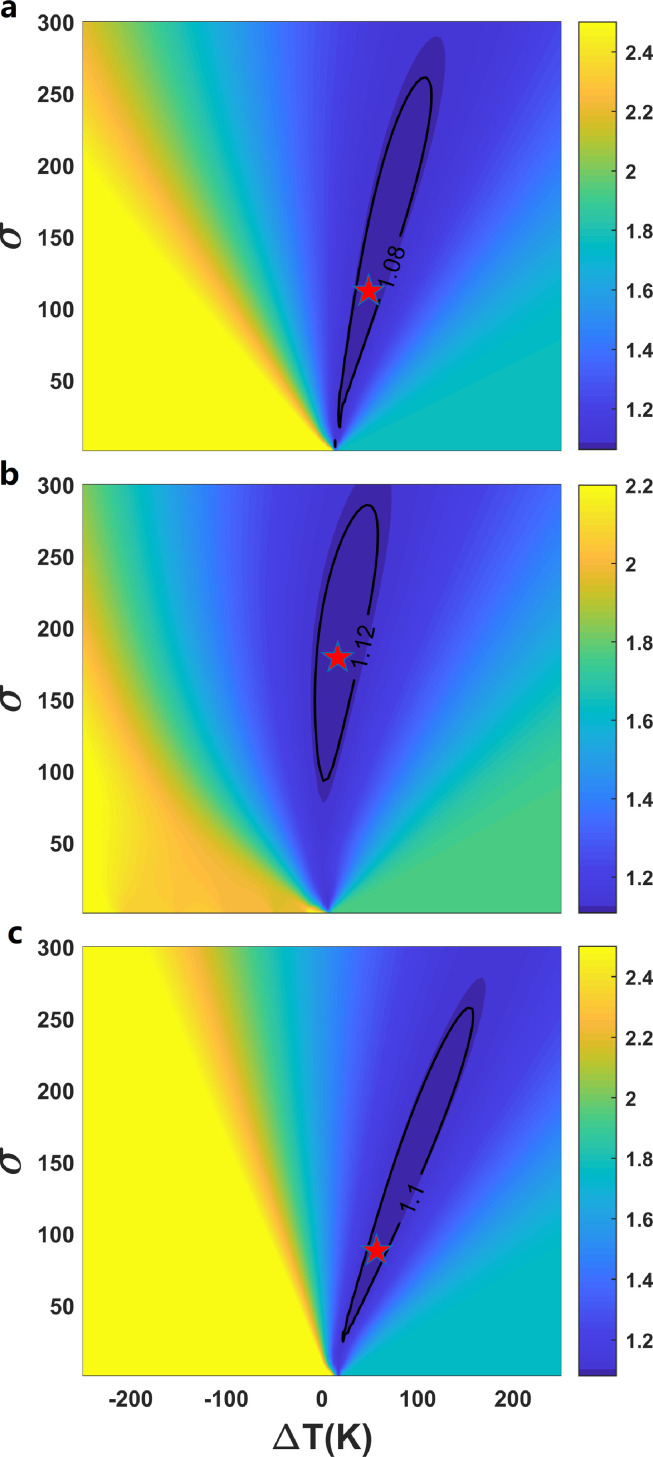
**The deviation**$D_{all}$**between pyrolite and AK135.** The results are based on phase boundary of (a) Yu et al. [19], (b) Ishii et al. [17], and (c) Hernández et al. [18] at different σ and ΔT. Stars are the points of the smallest $D_{all}$.

By assuming a Gaussian temperature distribution, we determined the volume proportion of akimotoite at the mantle geotherm $T_{g}$ with different standard deviations (σ is 0∼300) based on the MgSiO_3_ phase diagram (see Fig. 1a and Eq. 8). Combining elastic data of minerals in the lower MTZ (Table 2), we obtained the velocities and density of the pyrolite model. The calculated misfit function $D_{all}$ (Eq. 9) based on AK135 is shown in Fig. 6. ΔT is the temperature difference between $T_{g}$ and the triple point of MgSiO_3_ phase transition. The$\;D_{all}$ increases dramatically once ΔT<0K, suggesting that the triple point of the phase transition should be below the geotherm, which is consistent with the perspective that majorite is a stable phase along geotherm. The results from the conventional method, which ignores the temperature distribution, are represented by those withσ=0K. $D_{all}$ with σ=0Kfor the cases with ΔT>0K is ∼1.7% for AK135. Introducing the temperature distribution can reduce $D_{all}$ by ∼40%. The same conclusions can be derived with $D_{all}$ based on PREM (Fig. S3). As shown in Fig. 5, after considering the temperature distribution, the consistency between the velocities and density of the pyrolite model and those of PREM and AK135 at the depth range of 580-660 km is as good as or better than that at the depth range of 540-580 km. The harzburgite model contains only about 18.5 vol.% majoritic garnet. Introducing Gaussian distribution of temperature into the harzburgite model can only reduce $D_{all}$ ∼7% (Figs. S4 and S5), thus failing to match the velocities and density of PREM and AK135 at lower MTZ. Thus, our results demonstrate that the pyrolite model can explain well the velocities and density of the lower MTZ with temperature heterogeneity. The lower MTZ with a composition different from the upper mantle and lower mantle is not required.

### 3.3 The temperature distribution of the lower MTZ

The smallest $D_{all}^{AK135}$ occurs at ΔT=49K and σ=112K for Yu et al. [19], at ΔT=16K and σ=179K for Ishii et al. [17], and at ΔT=57K and σ=88K for Hernández et al. [18], respectively. The best σ and ΔT derived from PREM are larger than those from AK135. Wang et al. [11] constrained the water and temperature distribution at the base of MTZ by combining 660-km topography and seismic tomography. The σ from their temperature distribution is around 100 (Fig. 1b), which is closed to σ our fitted based on AK135. These results suggest that the temperature in the lower MTZ can be described by a Gaussian distribution with a σof∼100K and an average temperature of dozens of K higher than the triple point of MgSiO_3_. The conclusion is not sensitive to the iron partition coefficients K_D_ between akimotoite and ringwoodite (Table S4). The triple point of the MgSiO_3_ phase diagram provides another anchor point for the mantle geotherm.

For the lower MTZ, σ∼100K means that the volume proportions of mantle material with temperature 200 K lower than geotherm is ∼2.25%. The cold mantle is mainly caused by subducting slabs. Assuming that all subducting slabs penetrate into the lower mantle except the flat slabs which are stagnant above the 660 km discontinuity, using the subduction length, the dip angle of slab, the width of stagnant flat slab for each slab beneath arc volcanoes [61], [62], [63] and the petrological model of the slab with a thickness of 80 km [48], we calculated the volume proportion of subducting slabs in the lower MTZ globally, ∼2.88 vol.%. This may be overestimated since there are some young subducting slabs that have not been subducted to the lower MTZ yet. Considering only a fraction of the slab is 200 K lower than the ambient mantle, the estimation on volume proportion of slab is close to 2.25% in the lower MTZ and also supports that σ is ∼100K.

**Fig. 7:**
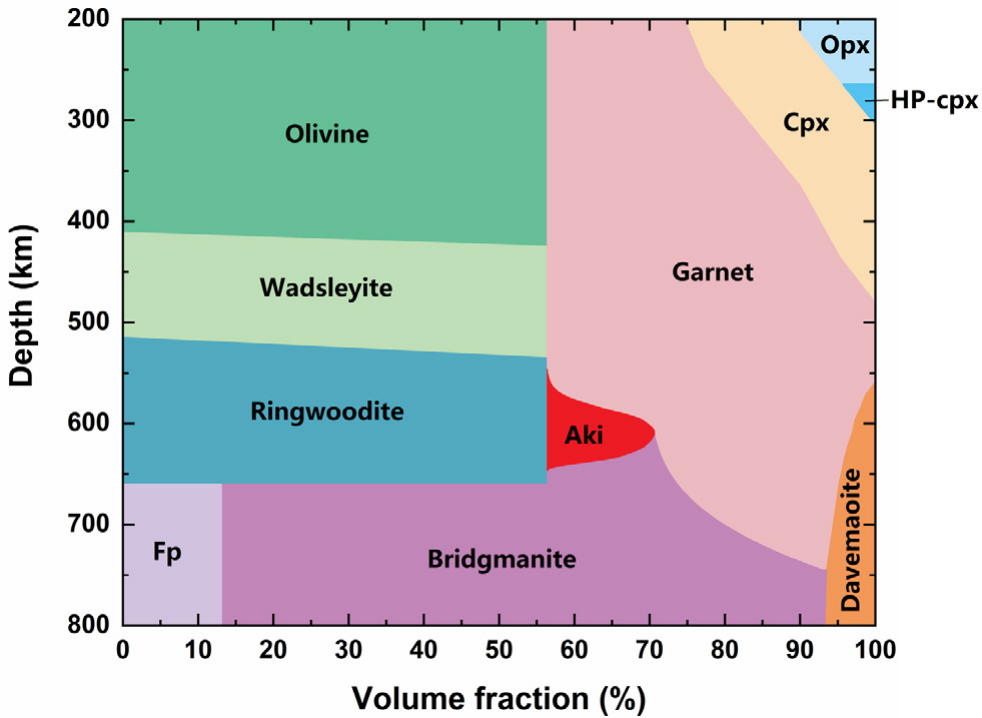
**The pyrolite model with temperature heterogeneity (modified from Frost****[46]****).** Opx = Orthopyroxene, HP-cpx = High-pressure clinopyroxene, Cpx = Clinopyroxene, Aki = Akimotoite, Fp = Ferropericlase. The phase boundary of orthopyroxene to high-pressure clinopyroxene is from Akashi et al. [69]. Although akimotoite is stable below the geotherm, the pyrolytic lower MTZ can contain a significant amount of akimotoite because of the temperature heterogeneity (Fig. 1). The velocities and density of the pyrolite model agree well with the seismic results in the lower MTZ after considering the temperature heterogeneity (Fig. 5)

The temperature distribution allows ∼10-15 vol.% akimotoite in the lower MTZ (Fig. 7). The strong elastic anisotropy of akimotoite (Fig. S1) provides a reasonable explanation for the anisotropy of the lower MTZ although ringwoodite and majoritic garnet, the major minerals in the lower MTZ, are almost isotropic. Global seismic observation shows that the radial anisotropy $\left(\xi =\frac{V_{SH}^{2}}{V_{SV}^{2}}\right)$ of the lower MTZ is less than 2.3% [64], [65], [66]. The anisotropy can be explained by no more than 5% akimotoite in the pyrolite model when the lattice of akimotoite is arranged in a preferred orientation completely. 10-15% akimotoite in the pyrolite model should be large enough to generate the weak anisotropy in lower MTZ. We updated the pyrolite model by considering the temperature heterogeneities (Fig. 7). The pyrolite model can well account for the seismic velocity profile in the lower MTZ. Thus, although the 660-km boundary partially blocks the circulation between the upper mantle and lower mantle as suggested by slab stagnant and 660-km topography at the short length scale [61,67,68] , a lower MTZ compositionally significantly different from the upper mantle and lower mantle is not a requisite. This is consistent with only part of slabs stagnant at the 660-km boundary. The 660-km boundary is not an efficient obstacle for mantle flow and the whole mantle convection is expected to be more favorable globally.

## 4 Conclusion

We investigate the elastic properties of akimotoite $\left(Mg_{0.875},Fe_{0.125}\right)\text{Si}O_{3}$ at high pressures and temperatures with first-principles calculations based on the density functional theory (DFT). The results are well consistent with available experimental data. In lower MTZ, the akimotoite is ∼20% (11%) larger in $V_{S}$ ($V_{P}$) than in majoritic garnet.

The volume proportion of akimotoite in a pyrolitic lower MTZ depends on the temperature heterogeneity. Combining the elasticity of akimotoite and other minerals in the MTZ, we estimate the velocity and density profiles of pyrolite with the Gaussian temperature distributions. We found that the velocities and density of pyrolite fit the seismic velocity profile well when the standard deviation of temperature for lower MTZ is ∼100 K and the geotherm locates at a little bit above the triple point of the MgSiO_3_ phase diagram. The triple point of the MgSiO_3_ phase diagram provides another anchor point for geotherm. The presence of ∼10-15 vol.% akimotoite at the base of MTZ provides a reasonable explanation for the anisotropy of the lower MTZ although the other major minerals in the lower MTZ are almost isotropic. A lower MTZ with composition significantly different from the pyrolite model is not a requisite and the whole mantle convection is expected to be more favorable globally.

## Declaration of Competing Interest

The authors declare that they have no conflicts of interest in this work.

## Appendix Supplementary materials

Image, application 1
