# Supplementary material for: Elastic properties of Fe-bearing Akimotoite at mantle conditions: Implications for composition and temperature in lower mantle transition zone
Source: Fundam Res. 2022 Jan 21;2(4):570–7. doi: 10.1016/j.fmre.2021.12.013 (PMC11197629; doi:10.1016/j.fmre.2021.12.013)
Supplement: Supplementary file 1 [file mmc1.docx]

**Elastic properties of Fe-bearing Akimotoite at mantle conditions: Implications for composition and temperature in lower mantle transition zone**

**Yajie Zhao^1^, Zhongqing Wu^1,2,3*^, Shangqin Hao^1,4^, Wenzhong Wang^5, 6^, Xin Deng^1^, Jian Song^1^**

^1^Laboratory of Seismic and Physics of Earth’s Interior, School of Earth and Space Sciences, University of Science and Technology of China, Hefei 230026, China.

^2^National Geophysical Observatory at Mengcheng, University of Science and Technology of China, Hefei, China.

^3^CAS Center for Excellence in Comparative Planetology, USTC, Hefei 230026, China.

^4^Institute of Geophysics and Planetary Physics, Scripps Institution of Oceanography, University of California San Diego, La Jolla 92092, CA, USA.

^5^Department of Earth Sciences, University College London, London WC1E 6BT, United Kingdom.

^6^Earth and Planets Laboratory, Carnegie Institution for Science, Washington, DC 20015, USA.

Corresponding author: Zhongqing Wu ([wuzq10@ustc.edu.cn](mailto:wuzq10@ustc.edu.cn))

**This PDF file includes:**

Supplementary text

Figures S1 to S6

Tables S1 to S4

SI References

Supplementary Information Text

The following supplementary materials provide the anisotropies of $\text{(}\text{Mg}_{\text{0.875}}\text{,}\text{Fe}_{\text{0.125}}\text{)Si}\text{O}_{\text{3}}$ akimotoite (Fig. S1), velocities and density contrasts between majorite and akimotoite, akimotoite and bridgmanite respectively (Fig. S2), the results of misfit function *D_all_* base on PREM (Fig. S3), test for sensitivity of the model to partition coefficients K_D_ between minerals (Fig. S4 and Table S4), model test for misfit function D_all_ between the harzburgite and seismic model (Fig. S5 and Fig. S6), the pressure and temperature dependences of elastic properties of $\text{(}\text{Mg}_{\text{0.875}}\text{,}\text{Fe}_{\text{0.125}}\text{)Si}\text{O}_{\text{3}}$ akimotoite (table S1-S3).

**Sensitivity of the model to partition coefficients K_D_**

The K_D_ of garnet-ringwoodite, akimotoite-ringwoodite, and ringwoodite-ferropericlase is not well constrained [1-6] (Fig. S4). We choose the average value of the reported K_D_ in the paper_._ The K_D_ influences the iron content of minerals and also the velocities and density of the pyrolite model. The difference for K_D_ of akimotoite-ringwoodite among the different group is relatively large (Fig. S4), mainly in 0.65-0.3. In order to check the sensitivity of the result to the K_D_, we test two extreme cases in which we choose the maximum (0.65) and minimum (0.3) K_D_ of akimotoite-ringwoodite, respectively. There is little variation in $D_{all}^{min}$ for PREM and AK135 (Table S4). Meanwhile, the $\sigma$ and $\Delta T$ are also similar for different K_D_.

**Model test for misfit function D_all_ between the harzburgite and seismic model**

The harzburgite model has been calculated by previous studies [7, 8]. The S-wave velocity of harzurgite model from Pamato et al. [7] is lower than PREM and AK135 significantly (Fig. S5). Siersch [8] calculated the harzburgite model which seems fitting the PREM and ak135 well after containing ~15% akimotoite (Fig. S5). The akimotoite is the necessary condition for fitting seismic models in the both pyrolite and harzburgite models calculation. Their results suggested a pure harzburgite layer in the lower MTZ. Though this condition is hardly to realize, and the mechanism of accumulating harzburgite is still controversial, we still test the deviation $D_{all}$ between harzburgite and AK135 (Fig. S6). As a whole, the $D_{all}$ between the harzburgite model and AK135 is larger than the deviation between pyrolite model and AK135. Along the normal mantle geotherm ($\sigma=0$), the $D_{all}$ between the harzburgite model and AK135 is ~1.41%. Introducing the Gaussian distribution of temperature can only reduce $D_{all}$ ~7%, which is far less than ~40% generated by introducing temperature heterogeneity to pyrolite model. The much less effect of temperature heterogeneity on harzburgite model than pyrolite model may be because harzburgite model contains only about 18.5 vol.% majoritic garnet. Based on above discussion, we prefer a pyrolytic lower MTZ with temperature heterogeneity.





Fig. S1. Pressure dependences of (a) $\boldsymbol{A}_{\boldsymbol{P}}$, (b) $\boldsymbol{A}_{\boldsymbol{S}}$ and (c) $\boldsymbol{A}_{\boldsymbol{S}}^{\boldsymbol{po}}$ at various temperatures. The solid lines are the anisotropies of (Mg_0.875,_Fe_0.125_)SiO_3_ in this study and the dashed lines are the anisotropies of MgSiO_3_ from Hao et al. [9].





Fig. S2. The velocities and density contrasts between majoritic garnet ((Mg_0.92,_Fe_0.08_)SiO_3_) and akimotoite ((Mg_0.94,_Fe_0.06_)SiO_3_), akimotoite ((Mg_0.94,_Fe_0.06_)SiO_3_) and bridgmanite ((Mg_0.9,_Fe_0.1_)SiO_3_) along the cold mantle geotherm (500 K colder than normal geotherm).


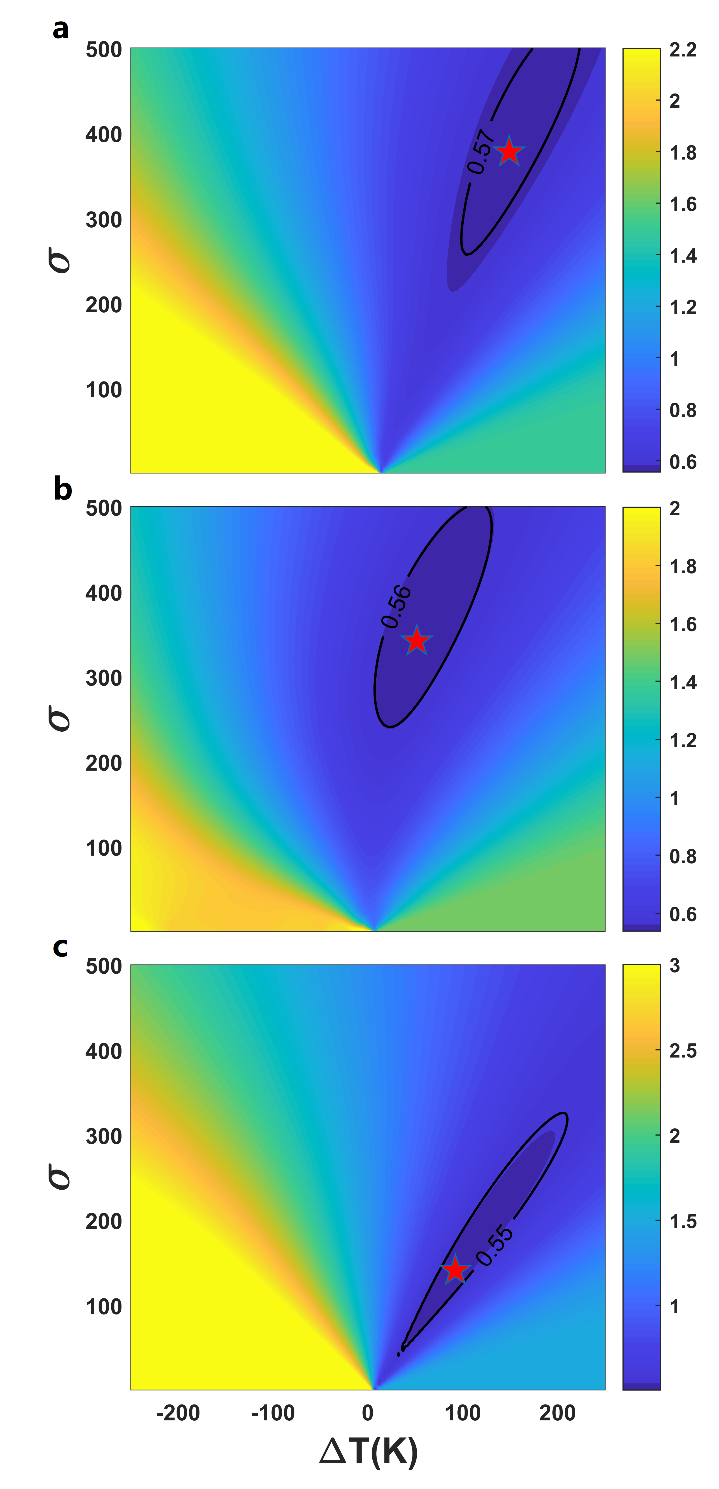


Fig. S3. The deviation $\boldsymbol{D}_{\boldsymbol{all}}$ between pyrolite and PREM. The results are based on phase boundary of (a) Yu et al. [10], (b) Ishii et al. [4], and (c) Hernández et al. [11] at different *σ* and $\boldsymbol{\Delta T}$. Stars are the points of smallest $\boldsymbol{D}_{\boldsymbol{all}}$.

**
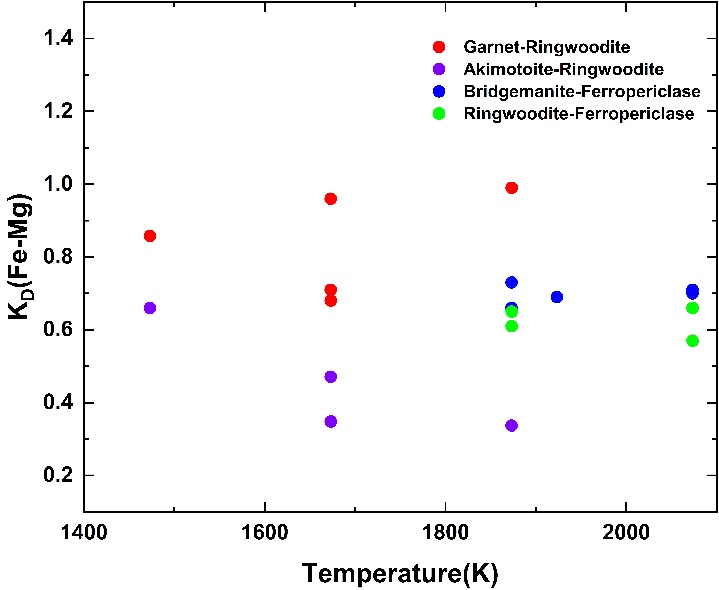
**

Fig. S4. The partition coefficients K_D_ of garnet-ringwoodite, akimotoite-ringwoodite, ringwoodite-ferropericlase, and bridgmanite-ferropericlase in previous studies [1-6].





Fig. S5. (a) Compressional wave velocities *V_P_*, (b) shear wave velocities *V_S_*, and (c) densities of the pyrolite, the harzburgite and the seismic models along the geotherm. The blue and green lines represent the harzburgite model without akimotoite and with 15% akimotoite, respectively [7, 8].


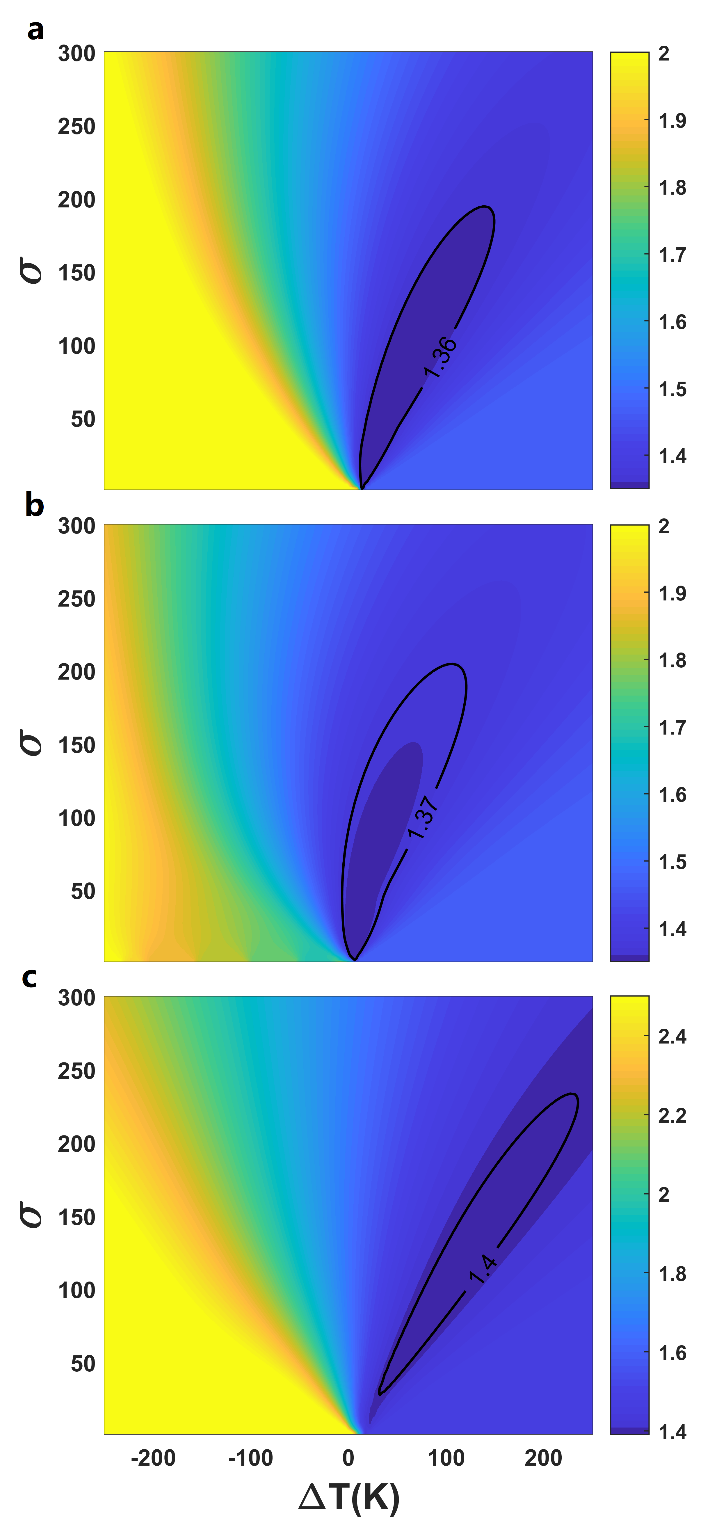


Fig. S6. The deviation 𝐷_𝑎𝑙𝑙_ between harzburgite and AK135. The results are based on phase boundary of (a) Yu et al. [12], (b) Ishii et al. [4], and (c) Hernández et al. [11] at different 𝜎 and ∆𝑇. Stars are the points of the smallest 𝐷_𝑎𝑙𝑙_.

Table S1. Fitting parameters of Elastic moduli with pressure of akimotoite at different temperatures.

| T (K) | C_11_ | C_12_ | C_13_ | C_33_ | C_44_ | C_14_ | C_25_ | K_S_ | G |
| --- | --- | --- | --- | --- | --- | --- | --- | --- | --- |
|  | M_0_ (GPa) | | | | | | | | |
| static | 451 | 155 | 94 | 361 | 103 | -15 | -26 | 214 | 126 |
| 300 | 436 | 150 | 86 | 346 | 96 | -16 | -23 | 204 | 121 |
| 300* |  |  |  |  |  |  |  | 197 | 121 |
| 1000 | 400 | 142 | 75 | 318 | 81 | -17 | -15 | 186 | 108 |
| 1500 | 369 | 135 | 65 | 294 | 68 | -17 | -8 | 171 | 96 |
| 2000 | 335 | 128 | 54 | 266 | 54 | -18 | 0 | 154 | 82 |
|  | $\partial M/\partial P$ | | | | | | | | |
| static | 5.64 | 3.29 | 4.05 | 5.48 | 2.11 | 0.25 | -0.58 | 4.42 | 1.53 |
| 300 | 6.00 | 3.30 | 4.10 | 5.81 | 2.27 | 0.24 | -0.64 | 4.57 | 1.71 |
| 300* |  |  |  |  |  |  |  | 5.2 | 1.5 |
| 1000 | 6.83 | 3.37 | 4.27 | 6.52 | 2.63 | 0.24 | -0.79 | 4.91 | 2.09 |
| 1500 | 7.53 | 3.37 | 4.38 | 7.17 | 2.96 | 0.24 | -0.95 | 5.18 | 2.46 |
| 2000 | 8.35 | 3.35 | 4.49 | 7.97 | 3.37 | 0.23 | -1.19 | 5.49 | 2.98 |
|  | ${\partial^{2}M}/{\partial P^{2}}$ ($\times{10}^{-3}$ GPa^-1^) | | | | | | | | |
| static | -18.66 | 1.25 | -5.24 | -29.59 | -13.88 | 0.46 | 4.51 | -10.26 | -12.97 |
| 300 | -20.74 | 2.01 | -5.34 | -32.33 | -15.31 | 0.51 | 5.42 | -10.86 | -14.46 |
| 1000 | -27.12 | 3.35 | -6.05 | -39.77 | -19.23 | 0.56 | 7.97 | -13.08 | -18.72 |
| 1500 | -32.92 | 5.17 | -6.35 | -47.08 | -23.15 | 0.64 | 10.75 | -14.94 | -23.45 |
| 2000 | -40.24 | 8.83 | -6.48 | -56.87 | -28.50 | 0.78 | 14.87 | -17.14 | -30.92 |

*M* represents the elastic moduli; *P* is pressure in GPa. The data are fitted for pressure range of 0-30 GPa based on the equation: $M=M_{0}+(\partial M/\partial P)\times P+{(\partial^{2}M}/{\partial P^{2})}\times P^{2}$. 300* is the data from Siersch [8].

Table S2. Fitting parameters of Elastic moduli with temperature of akimotoite at different pressures.

| P  (GPa) | C_11_ | C_12_ | C_13_ | C_33_ | C_44_ | C_14_ | C_25_ | K_S_ | G |
| --- | --- | --- | --- | --- | --- | --- | --- | --- | --- |
|  | M_0_ (GPa) | | | | | | | | |
| 0 | 436 | 150 | 87 | 346 | 96 | -16 | -22 | 204 | 120 |
| 10 | 494 | 183 | 127 | 402 | 117 | -13 | -28 | 249 | 136 |
| 20 | 547 | 217 | 167 | 450 | 135 | -11 | -33 | 292 | 149 |
| 30 | 597 | 251 | 205 | 492 | 151 | -8 | -37 | 332 | 159 |
|  | $\partial M/\partial T$ (MPa/K) | | | | | | | | |
| 0 | -46.51 | -11.53 | -15.94 | -35.83 | -18.84 | -1.25 | 10.18 | -23.69 | -15.61 |
| 10 | -36.78 | -9.95 | -13.41 | -27.98 | -15.05 | -1.26 | 9.04 | -19.40 | -12.08 |
| 20 | -28.75 | -8.21 | -11.51 | -20.02 | -12.21 | -1.26 | 8.39 | -15.76 | -9.19 |
| 30 | -21.94 | -6.42 | -9.74 | -17.25 | -9.99 | -1.27 | 8.07 | -12.58 | -6.83 |
|  | ${\partial^{2}M}/{\partial T^{2}}$ ($\times{10}^{-6}$ GPa/K^2^) | | | | | | | | |
| 0 | -7.73 | 0.69 | -1.98 | -7.18 | -4.10 | -0.05 | 1.81 | -3.51 | -4.43 |
| 10 | -5.86 | -1.47 | -1.94 | -4.83 | -2.30 | -0.09 | 0.76 | -2.99 | -2.41 |
| 20 | -4.61 | -1.67 | -1.91 | -3.61 | -1.65 | -0.10 | 0.31 | -2.60 | -1.62 |
| 30 | -3.72 | -1.70 | -1.80 | -2.86 | -1.25 | -0.11 | 0.08 | -2.31 | -1.19 |

*M* represents the elastic moduli; *T* is temperature in Kelvin. The data are fitted for the temperature range of 270-2000 K based on the equation: $M=M_{0}+(\partial M/\partial T)\times(T-300)+{(\partial^{2}M}/{\partial T^{2})}\times{(T-300)}^{2}$*.*

Table S3. Wave velocities and their first and second derivatives with respect to pressures and temperatures of akimotoite.

| P (GPa) | $V_{P}$ (km/s) | $\partial V_{P}/\partial T$ ($\times{10}^{-3}$ km/s/K) | ${\partial^{2}V_{P}}/{\partial T^{2}}$ ($\times{10}^{-8}$ km/s/K^2)^ | $V_{S}$ (km/s) | $\partial V_{S}/\partial T$ ($\times{10}^{-3}$ km/s/K) | ${\partial^{2}V_{S}}/{\partial T^{2}}$ ($\times{10}^{-8}$ km/s/K^2)^ |
| --- | --- | --- | --- | --- | --- | --- |
| 0 | 9.67 | -0.429 | -16.460 | 5.55 | -0.254 | -14.050 |
| 10 | 10.28 | -0.307 | -8.365 | 5.78 | -0.187 | -6.044 |
| 20 | 10.75 | -0.212 | -5.270 | 5.93 | -0.129 | -3.383 |
| 30 | 11.15 | -0.139 | -3.639 | 6.03 | -0.084 | -2.112 |
| T (K) | $V_{P}$ (km/s) | $\partial V_{P}/\partial P$ ($\times{10}^{-2}$ km/s/GPa) | ${\partial^{2}V_{P}}/{\partial P^{2}}$ ($\times{10}^{-4}$ km/s/GPa^2)^ | $V_{S}$ (km/s) | $\partial V_{S}/\partial P$ ($\times{10}^{-2}$ km/s/GPa) | ${\partial^{2}V_{S}}/{\partial P^{2}}$ ($\times{10}^{-4}$ km/s/GPa^2)^ |
| static | 9.83 | 5.819 | -4.577 | 5.65 | 2.033 | -2.615 |
| 300 | 9.68 | 6.432 | -5.244 | 5.56 | 2.464 | -3.104 |
| 1000 | 9.29 | 7.859 | -7.051 | 5.31 | 3.483 | -4.497 |
| 1500 | 8.93 | 9.287 | -9.041 | 5.06 | 4.590 | -6.189 |
| 2000 | 8.47 | 11.270 | -12.110 | 4.73 | 6.269 | -9.051 |

The pressure derivatives are fitted for the range of 0-30 GPa based on the equation: $V=V_{0}+(\partial V/\partial P)\times P+{(\partial^{2}V}/{\partial P^{2})}\times P^{2}$, and the temperature derivatives for the range of 270-2000 K are based on $V=V_{0}+(\partial V/\partial T)\times(T-300)+{(\partial^{2}V}/{\partial T^{2})}\times{(T-300)}^{2}$, where *V* represents *V_P_* or *V_S_*; *P* is pressure in GPa; *T* is temperature in Kelvin.

Table S4. Results from Different K_D_ of akimotoite-ringwoodite.

| Model | K_D_ | Phase diagram | $\boldsymbol{D}_{\boldsymbol{all}}^{\boldsymbol{min}}$ | sigma | $\boldsymbol{\Delta T}$ |
| --- | --- | --- | --- | --- | --- |
| AK135 | 0.3 | Yu2011 | 1.06 | 144 | 19 |
|  |  | Ishii2011 | 1.12 | 175 | 22 |
|  |  | Hernández2015 | 1.09 | 86 | 57 |
| AK135 | 0.65 | Yu2011 | 1.03 | 143 | 54 |
|  |  | Ishii2011 | 1.06 | 197 | 2 |
|  |  | Hernández2015 | 1.04 | 103 | 62 |
| PREM | 0.3 | Yu2011 | 0.54 | 429 | 149 |
|  |  | Ishii2011 | 0.52 | 364 | 37 |
|  |  | Hernández2015 | 0.49 | 160 | 102 |
| PREM | 0.65 | Yu2011 | 0.56 | 364 | 149 |
|  |  | Ishii2011 | 0.54 | 336 | 56 |
|  |  | Hernández2015 | 0.51 | 140 | 92 |

**SI References**

[1] Y. Zhang, Y. Wang, Y. Wu, C.R. Bina, Z. Jin, S. Dong, Phase transitions of harzburgite and buckled slab under eastern China, Geochemistry, Geophysics, Geosystems, 14 (2013) 1182-1199, <https://doi.org/10.1002/ggge.20069>.

[2] T. Ishii, H. Kojitani, M. Akaogi, Phase relations and mineral chemistry in pyrolitic mantle at 1600–2200 °C under pressures up to the uppermost lower mantle: Phase transitions around the 660-km discontinuity and dynamics of upwelling hot plumes, Physics of the Earth and Planetary Interiors, 274 (2018) 127-137, <https://doi.org/10.1016/j.pepi.2017.10.005>.

[3] K. Hirose, Phase transitions in pyrolitic mantle around 670-km depth: Implications for upwelling of plumes from the lower mantle, Journal of Geophysical Research: Solid Earth, 107 (2002) ECV 3-1-ECV 3-13, <https://doi.org/10.1029/2001jb000597>.

[4] T. Ishii, H. Kojitani, M. Akaogi, Post-spinel transitions in pyrolite and Mg2SiO4 and akimotoite–perovskite transition in MgSiO3: Precise comparison by high-pressure high-temperature experiments with multi-sample cell technique, Earth and Planetary Science Letters, 309 (2011) 185-197, <https://doi.org/10.1016/j.epsl.2011.06.023>.

[5] D. Frost, F. Langenhorst, P. Van Aken, Fe–Mg partitioning between ringwoodite and magnesiowüstite and the effect of pressure, temperature and oxygen fugacity, Physics and Chemistry of Minerals, 28 (2001) 455-470, <https://doi.org/10.1007/s002690100181>.

[6] D.J. Frost, F. Langenhorst, The effect of Al2O3 on Fe–Mg partitioning between magnesiowüstite and magnesium silicate perovskite, Earth and Planetary Science Letters, 199 (2002) 227-241, <https://doi.org/10.1016/S0012-821X(02)00558-7>.

[7] M.G. Pamato, A. Kurnosov, T. Boffa Ballaran, D.J. Frost, L. Ziberna, M. Giannini, S. Speziale, S.N. Tkachev, K.K. Zhuravlev, V.B. Prakapenka, Single crystal elasticity of majoritic garnets: Stagnant slabs and thermal anomalies at the base of the transition zone, Earth and Planetary Science Letters, 451 (2016) 114-124, <https://doi.org/10.1016/j.epsl.2016.07.019>.

[8] N.C. Siersch, The effect of Fe and Al on the elasticity of akimotoite, (Doctoral dissertation), (2019), <https://doi.org/10.15495/EPub_UBT_00004577>.

[9] S. Hao, W. Wang, W. Qian, Z. Wu, Elasticity of akimotoite under the mantle conditions: Implications for multiple discontinuities and seismic anisotropies at the depth of ∼600–750 km in subduction zones, Earth and Planetary Science Letters, 528 (2019) 115830, <https://doi.org/10.1016/j.epsl.2019.115830>.

[10] Y.G. Yu, R.M. Wentzcovitch, V.L. Vinograd, R.J. Angel, Thermodynamic properties of MgSiO3majorite and phase transitions near 660 km depth in MgSiO3and Mg2SiO4: A first principles study, Journal of Geophysical Research, 116 (2011), <https://doi.org/10.1029/2010jb007912>.

[11] E.R. Hernández, J. Brodholt, D. Alfè, Structural, vibrational and thermodynamic properties of Mg2SiO4 and MgSiO3 minerals from first-principles simulations, Physics of the Earth and Planetary Interiors, 240 (2015) 1-24, <https://doi.org/10.1016/j.pepi.2014.10.007>.

[12] Y.G. Yu, R.M. Wentzcovitch, V.L. Vinograd, R.J. Angel, Thermodynamic properties of MgSiO3 majorite and phase transitions near 660 km depth in MgSiO3 and Mg2SiO4: A first principles study, Journal of Geophysical Research, 116 (2011), <https://doi.org/10.1029/2010jb007912>.
